# The importance of structural, situational, and psychological factors for involving hunters in the adaptive flyway management of geese

**DOI:** 10.1038/s41598-023-33846-0

**Published:** 2023-05-02

**Authors:** Louise Eriksson, Johan Månsson, Niklas Liljebäck, Camilla Sandström, Maria Johansson, Ann Eklund, Johan Elmberg

**Affiliations:** 1 Department of Geography, Umeå University, 901 87 Umeå, Sweden; 2 Grimsö Wildlife Research Station, Department of Ecology, Swedish University of Agricultural Sciences, 730 91 Riddarhyttan, Sweden; 3 Department of Political Science, Umeå University, 901 87 Umeå, Sweden; 4 Environmental Psychology, Department of Architecture and Built Environment, Lund University, 221 00 Lund, Sweden; 5 Department of Environmental Science and Bioscience, Kristianstad University, 291 88 Kristianstad, Sweden

**Keywords:** Environmental social sciences, Psychology and behaviour

## Abstract

Adaptive flyway management of superabundant geese is emerging as a strategy to reduce damage to agricultural crops and other ecosystem disservices, while also ensuring sustainable use and conservation objectives. Given the calls for intensified hunting as part of flyway management in Europe, we need to increase the understanding of structural, situational, and psychological factors important for goose hunting among hunters. Our survey data, retrieved in southern Sweden, showed a higher potential to intensify hunting among goose hunters than other hunters. In response to hypothetical policy instruments (including regulations, collaborative, and others), hunters declared a minor increase in their intention to hunt geese, with the greatest expected increase among goose hunters should the hunting season be extended. Situational factors (e.g., access to hunting grounds) were associated with goose hunting (frequency, bag size, and intention to increase hunting). In addition, controlled motivation (derived from external pressures or to avoid guilt) and more importantly autonomous motivation (due to hunting being enjoyable or valuable) were along with goose hunter identity positively associated with goose hunting. Hunters’ involvement in flyway management may be encouraged by using policy instruments to remove situational barriers and facilitate their autonomous motivation.

## Introduction

Management of geese and other migratory waterbirds is challenging as they move over large areas, and across national borders with different legislation, cultures, and norms. For geese, some species are rare and in need of conservation efforts while other species have gone from globally threatened to superabundant with negative impact on ecosystems and human interests (e.g., crop damage and air safety concerns) leading to widespread conflict among stakeholders^1–4^.

Flyway management plans, resting on the principles of adaptive management^5,6^, have been endorsed to manage damage concerns and conflicts, while ensuring conservation efforts, sustainable use, and population regulation depending on the status of the species^7–10^. Implementation of flyway management of geese involves a multitude of stakeholder groups (e.g., farmers, conservationists, and hunters) and includes tools such as habitat improvement, monitoring, scaring, and hunting^11–13^. In particular hunters are considered to have a stewardship role in population regulation. Historically, overharvest and population decline of many goose populations were in focus of waterbird management and more strict regulations of hunting were regarded as a successful conservation intervention allowing recovery of small populations^4^. Conversely, goose management in North America and Europe now face the challenge to increase harvest of superabundant populations^2–4,14^. Given the recent changes in management objectives, an interest in instruments to intensify goose hunting effort as well as making hunting more effective has been noted in Europe^15,16^. Intensifying hunting may involve increased hunting effort during open hunting season, as well as derogation shooting for damage control of both protected and huntable species outside the open hunting season. Since the implementation of adaptive flyway management of geese is dependent on hunters taking on a stewardship role, such management requires an understanding of the drivers and barriers of hunting. How to recruit new or lapsed goose hunters and how to keep them engaged have been examined predominantly in North America, highlighting the importance of socialization and the need to remove personal barriers and conditions interfering with the possibilities to hunt^17–21^. Nevertheless, there is still a gap in the understanding of what factors are associated with increasing hunters’ effort and realizing larger hunting bags, despite it being critical to reach outlined management objectives. This study examines structural, situational, and psychological factors associated with goose hunting, including the possibility for increased goose hunting among hunters in Sweden.

### Drivers and barriers of hunting

Studies examining drivers and barriers of hunting (e.g., hunting frequency, bag size) commonly identify explicit, or stated, hunting motives related to nature or escaping from everyday life, social inclusion, achievement or excitement, and game meat^20,22–25^. In addition, hunting can provide collective gains for management^26,27^. However, motives to hunt geese (e.g., for meat or the challenge of hunting) were not important for goose bag size in a study of Danish hunters^28^. In addition, the use of explicit motives to determine hunting satisfaction (potentially associated with a desire to continue hunting)^29^ has been questioned^23^.

Barriers to continue or start hunting have been mapped extensively, though more so in the North American context than elsewhere. Such barriers comprise lack of interest, perceived lack of knowledge and skills, lack of time and financial resources, limited access to hunting grounds, low game density, low social acceptance, and too many regulations^17–21,26,28,30^. Older age and female gender have been found to be associated with non-hunting in these studies. Lack of knowledge/skills and lower access to multiple hunting grounds are identified potential barriers for goose hunting in a European context^15,28^.

Key barriers may be targeted by various policy instruments to ultimately have an impact on hunting and goose management performance^31^. Such instruments are of different types, including regulatory (e.g., changed hunting season, bag limits, ban on lead shot, report requirements, management zones), financial (subsidies and fees), collaborative (e.g., voluntary hunting agreements), educational, and various strategies to make hunting more accessible^16,22,30,32–34^. A longer hunting season has been shown to increase the total annual goose harvest by individual Danish hunters^32^. Voluntary agreements featuring hunting-free days and safe foraging areas have further been shown to increase goose presence, resulting in increased hunting success^16^. Hunters’ responses to policy instruments have been examined, e.g., attitudes towards regulations on waterbird hunting^22,33,35^, but such studies do not necessarily reflect how hunters would act if a change in policy were to be implemented. Hence, there is a need to also consider responses to policy that more closely reflect behaviors.

### Conceptual framework

**Figure 1:**
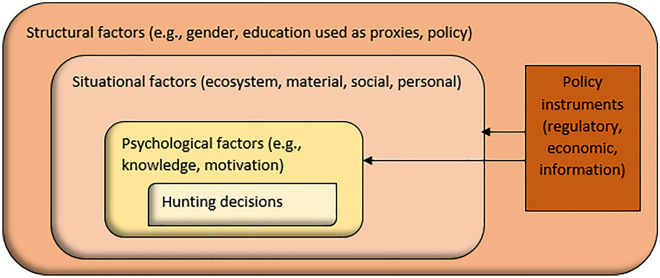
Conceptual framework for how a socio-ecological structure with factors at each level (structural, situational, and psychological) influence hunting decisions (e.g., what to hunt, how often).

Individuals’ decision to hunt can be considered the outcome of psychological processes within a social-ecological structure^36^. Closest to the individual are family, friends, and mentors, followed by the local community and surrounding landscape, and finally the broader society. *Structural factors* (e.g., gender and education used as proxies, policy) create a societal context for hunting practices. Hunting decisions and behaviors are furthermore influenced by *situational factors*, including the ecosystem (e.g., availability of game), material (e.g., access to hunting grounds, equipment), social (e.g., what close others do themselves and say are acceptable behaviors, i.e., descriptive and injunctive social norms, respectively), and personal (e.g., time) dimensions. Finally, *psychological factors* (e.g., knowledge, motivation) are important for hunters’ decisions, including the hunters’ beliefs of the actual situation, thereby supporting a need to also understand these beliefs^17,21,37^. Consequently, policy instruments can be used to target situational and/or psychological factors with an impact on hunting decisions (Fig. 1).

**Figure 2:**
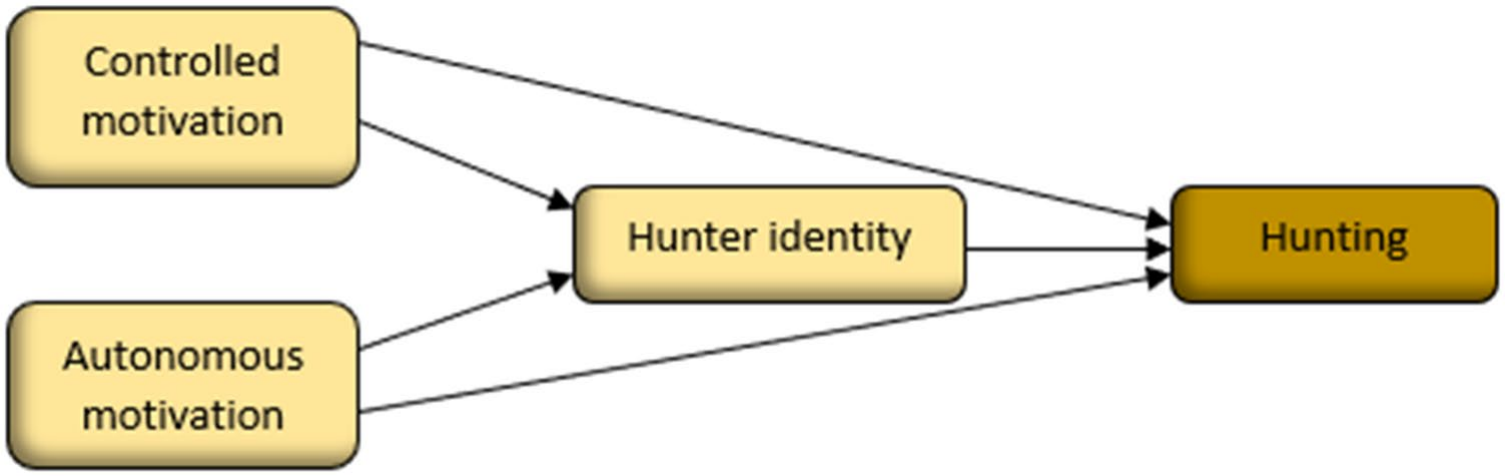
Controlled motivation (due to external pressures or to avoid guilt) and autonomous motivation (because hunting is enjoyable or valuable) as predictors of hunting, via hunter identity.

Given hunters’ position in a social-ecological structure, hunting decisions and subsequent hunting behaviors can be considered based on a combination of external and internal motivation^38,39^. The present study integrates self-determination theory (SDT) of motivation^40^ with identity theory^41^ in a novel approach to understand motivations for hunting (Fig. 2), thereby providing a theoretically based approach to study motivation. According to the SDT, behaviors vary in the extent to which they are externally versus internally motivated. *Controlled* motivation is driven from external demands such as situational rewards and punishments or from a desire to avoid e.g., guilt. Motivations that are increasingly originating from within the self, i.e., more internalized, are labelled *autonomous* motivation, driven by the inherent desire to conduct the activity, to ensure alignment with who they are, or personal importance. More autonomous forms of motivation have been found to be associated with e.g., personal growth and well-being.

Identity theory further illustrates how conceptions of the self (self-identities)^42^ can be associated with a role or group (e.g., hunter) and vary in importance^43,44^. Individuals have a desire to act in accordance with their self-identity and studies have confirmed the importance of hunter identity (i.e., the extent to which they identify as a hunter) for behaviors^41,45–47^. An integration of SDT and identity concepts proposed by Wang et al.^48^ suggests that controlled and autonomous motivation are important for identity, which in turn is important for behavioural intentions. More autonomous motivation is further associated stronger with self-identity than controlled motivation^49^.

### The present study

This survey study examined drivers and barriers of goose hunting and the potential to increase it in a representative sample of hunters in the south of Sweden. We grouped hunters depending on their experience of goose hunting and analyzed how different characteristics are associated with goose hunting. First, hunting in general and structural characteristics (e.g., gender) of the groups were compared. In addition, comparisons were made regarding hunters’ positive and negative beliefs about goose hunting reflecting situational and psychological factors. Second, the potential for increased goose hunting effort was analyzed by comparing intention to increase goose hunting and expectations regarding a decrease or increase in goose hunting in response to a set of hypothetical policy instruments (collaboration, education, financial, extended hunting season, and ways of making goose hunting more accessible). Third, the importance of situational factors (e.g., access to hunting grounds), motivation (controlled, autonomous) and goose hunter identity for current goose hunting (frequency and bag size), and intention to increase goose hunting was examined. An integration of SDT and self-identity stipulates that controlled and autonomous motivation are important for goose hunting, but autonomous motivation may have mainly an indirect effect via goose hunter identity. This is likely since more internalized motivation can be argued to facilitate a stronger goose hunter identity, which in turn would boost goose hunting (cf.^48,49^).

## Results

### Hunting in general and structural characteristics

Differences between ‘Non-goose hunters’ (n = 675), ‘Lapsed goose hunters’ (n = 278), and ‘Goose hunters’ (n = 606) regarding general hunting behavior and structural characteristics were generally small, but ‘Goose hunters’ displayed a much higher level of general hunting activity compared to the ‘Non-goose hunters’ and ‘Lapsed goose hunters’ (Table 1). Whereas the ‘Goose hunters’ and the ‘Non-goose hunters’ had started to hunt about the same number of years ago, ‘Lapsed goose hunters’ had started to hunt earlier. Having a parent involved in goose hunting was most common among ‘Goose hunters’, followed by ‘Lapsed goose hunters’ and least common among ‘Non-goose hunters’. Among ‘Goose hunters’ a larger share were men, fewer had a university degree, and a larger share lived in a rural area compared to ‘Non-goose hunters’, but the two groups were about the same age. The ‘Lapsed goose hunters’ were structurally similar to ‘Goose hunters’, but slightly older.

**Table 1:** General hunting behavior and structural characteristics in three groups of hunters.

	Non-goose hunters (N = 675)	Lapsed goose hunters (N = 278)	Goose hunters (N = 606)	Effect size	
				Cramers’ V	Partial η^2^
**General hunting behavior**					
Started hunting					
0–10 years ago	33%	8%	26%***	0.18	na
11–20 years ago	17%	12%	17%		
21–30 years ago	19%	21%	20%		
31 years or more ago	31%	59%	38%		
Hunting activity in 2021					
No	11%	8%	2%***	0.24	na
1–10 days	42%	28%	21%		
11–20 days	23%	23%	22%		
21–50 days	18%	28%	38%		
More than 50 days	6%	13%	19%		
**Structural characteristics**					
Parent was a goose hunter	5%	22%	27%***	0.28	na
Gender	86% men	94% men	94% men***	0.13	na
Age	50 years^a^	53 years^b^	49 years^a^	na	0.02
University degree	47%	37%	36%***	0.10	na
Rural residence	65%	73%	71%*	0.07	na

Means having the same superscript letter did not differ at *p* < 0.05 (ANOVA with Bonferroni correction). Following guidelines proposed by Cohen^65^, a small, medium, and large effect size correspond to Cramer’s V = 0.1–0.3, Cramer’s V = 0.3–0.5, and Cramer’s V > 0.5 for 1 df (i.e., parent was a goose hunter, gender, university degree, and rural residence), Cramer’s V = 0.07–0.21, Cramer’s V = 0.21–0.35, and Cramer’s V > 0.35 for 2 df (i.e., started hunting, hunting activity in 2021), and Partial η^2^ = 0.01, Parital η^2^ = 0.06, and Partial η^2^ = 0.14 (i.e., age), respectively.

*p < 0.05; ***p < 0.001. na = non-applicable.

### Situational and psychological characteristics associated with goose hunting

Group differences in beliefs about situational and psychological characteristics associated with goose hunting varied with the greatest differences found for beliefs reflecting limited knowledge as well as considering goose hunting to be prevalent and part of the local culture (i.e., descriptive goose hunting norm) (Table 2). Compared to the other groups, ‘Goose hunters’ displayed stronger positive beliefs about goose hunting and placed less emphasis on its negative aspects, except that goose hunting was believed to be time consuming. Furthermore, ‘Goose hunters’ experienced that other hunters are involved in goose hunting, reflecting a descriptive goose hunting norm. Results revealed that ‘Non-goose hunters’ strongly believed that they had limited knowledge of goose hunting. All hunter groups believed that goose hunting is highly accepted among their peers, indicating that injunctive goose hunting norms are less important to differentiate between the groups. Not surprisingly, ‘Goose hunters’ were more inclined to display beliefs that goose hunting is more exciting than the other groups. ‘Non-goose hunters’ held stronger beliefs that goose hunting is boring, but also that the number of geese is too low, compared to the other hunters. Differences between the groups were minor for acceptance of goose hunting in the general public, poor access to equipment, and believing that goose hunting is demanding. Overall, group differences were most pronounced in relation to psychological and social characteristics and less so for e.g., material and personal dimensions.

**Table 2:** Positive and negative beliefs about goose hunting reflecting situational and psychological characteristics in three groups of hunters.

	Non-goose hunters (N = 675)	Lapsed goose hunters (N = 278)	Goose hunters (N = 606)	Effect size
	M (SD)	M (SD)	M (SD)	Partial η^2^
Positive beliefs				
Part of hunting culture (S)	1.92 (1.08)^c^	2.47 (1.18)^b^	3.22 (1.33)^a^	0.19
Exciting (Ps)	3.24 (1.11)^c^	3.50 (1.15)^b^	4.10 (1.00)^a^	0.12
Easy access to hunting grounds (M)	2.17 (1.07)^b^	2.32 (1.22)^b^	2.71 (1.30)^a^	0.04
Accepted among people around me (S)	4.17 (1.01)^b^	4.30 (0.92)^b^	4.53 (0.77)^a^	0.03
Accepted in the general public (S)	3.43 (1.01)^b^	3.49 (1.05)^a,b^	3.62 (0.98)^a^	> 0.01
Negative beliefs				
Limited knowledge (Ps)	4.07 (1.05)^a^	3.19 (1.11)^b^	2.54 (1.14)^c^	0.29
Goose hunting not prevalent where I live (S)	3.95 (1.17)^a^	3.40 (1.26)^b^	2.68 (1.26)^c^	0.18
Boring (Ps)	2.50 (1.15)^a^	2.17 (1.06)^b^	1.71 (0.97)^c^	0.10
Too few geese (E)	2.72 (1.37)^a^	2.50 (1.36)^b^	1.94 (1.19)^c^	0.07
Time consuming (P)	2.90 (1.04)^c^	3.09 (1.17)^b^	3.47 (1.11)^a^	0.05
Poor access to retriever dog (M)	3.13 (1.26)^a^	2.97 (1.26)^a^	2.62 (1.37)^b^	0.03
Poor access to equipment (M)	2.67 (1.21)^a^	2.43 (1.16)^b^	2.54 (1.25)^a,b^	> 0.01
Demanding (P)	2.81 (1.02)^a^	2.56 (1.07)^b^	2.68 (1.15)^a,b^	> 0.01

*E* ecosystem, *M* material, *S* social, *P* personal, *Ps* psychological.

Means having the same superscript letter did not differ at *p* < 0.05 (ANOVA with Bonferroni correction). Following guidelines proposed by Cohen^65^, a small, medium, and large effect size correspond to Partial η^2^ = 0.01, Partial η^2^ = 0.06, and Partial η^2^ = 0.14.

### Potential for increased goose hunting

Results revealed large differences in intention to increase goose hunting among the groups with ‘Goose hunters’ displaying the strongest intention but still not exceeding the mid-point of the scale (Table 3). In addition, the three groups of hunters expected that they would increase goose hunting in response to policy instruments, but to a limited extent. ‘Goose hunters’ expected that they would increase goose hunting in response to an extended hunting season and financial instruments slightly more than would the other groups. In contrast, ‘Non-goose hunters’ and ‘Lapsed goose hunters’ expected that they would increase goose hunting slightly more in response to collaborative instruments compared to ‘Goose hunters’. Both ‘Goose hunters’ and ‘Non-goose hunters’ expected a greater increase in response to educational instruments compared to ‘Lapsed goose hunters’. There were no differences in how the groups responded to the possibilities for borrowing equipment and improving access to game meat processing plants.

**Table 3:** Intention to increase goose hunting and expected response to policy instruments in three groups of hunters.

	Non-goose hunters (N = 675)	Lapsed goose hunters (N = 278)	Goose hunters (N = 606)	Effect size
	M (SD)	M (SD)	M (SD)	Partial η^2^
Intention to increase goose hunting	1.76 (0.92)^c^	1.97 (0.97)^b^	2.90 (1.03)^a^	0.23
Response to policy instruments				
Extended hunting season	3.33 (0.79)^b^	3.37 (0.93)^b^	3.86 (0.89)^a^	0.08
Financial instruments	3.31 (0.84)^b^	3.32 (0.98)^b^	3.60 (0.96)^a^	0.02
Collaborative instruments	3.31 (0.86)^a^	3.24 (0.91)^a^	3.05 (0.96)^b^	0.02
Educational instruments	3.37 (0.83)^a^	3.19 (0.96)^b^	3.36 (0.91)^a^	0.01
Borrow equipment	3.24 (0.78)	3.19 (0.90)	3.31 (0.78)	> 0.01
Access to game meat processing plants	3.19 (0.82)	3.14 (1.04)	3.21 (1.02)	> 0.01

Means having the same superscript letter did not differ at *p* < 0.05 (ANOVA with Bonferroni correction). Following guidelines proposed by Cohen^65^, a small, medium, and large effect size correspond to Partial η^2^ = 0.01, Partial η^2^ = 0.06, and Partial η^2^ = 0.14.

### Drivers of goose hunting

Among ‘Goose hunters’, about one third (36%) went goose hunting during the open hunting season and for derogation on average 1–5 days, 29% for 6–10 days and 27% for 11 days or more (Table S2). Moreover, 14% had an average bag size during a goose hunt of 0–2 geese, 41% 3–5 geese, 24% 6–10 geese and 21% more than 11 geese. It was most common to get access to hunting ground by being invited as a hunting guest, followed by oral and leasing agreements (Table S3). The majority (68%) of ‘Goose hunters’ had access to either one or two hunting grounds, and 76% had less than 40 km to their most frequently used hunting ground. About one quarter (23%) had a dog in the household for goose hunting purposes. Only 15% did not have any goose hunter among family and friends, but 65% had at least a few goose hunters, and the remaining had more than that. ‘Goose hunters’ displayed stronger autonomous motivation (M = 3.12, SD = 0.86) than controlled motivation (M = 1.77, SD = 0.60), and overall, a weak goose hunter identity (M = 2.43, SD = 0.83).

There was no evidence of collinearity in the three hierarchical regression models examining determinants of goose hunting with the highest VIF values around 1.500 (1.494, 1.461, and 1.502, respectively). The first step in the regression models revealed that a similar set of situational determinants was associated with the different indicators (Table 4). More specifically, greater access to hunting ground by oral agreement, access to more hunting grounds, having a dog in the household for goose hunting purposes, and having more goose hunters among family and friends were associated with a greater hunting frequency and a larger bag size. Distance to the most frequently used hunting ground was negatively associated only with hunting frequency and owning a hunting ground was negatively associated with bag size only. The situational determinants remained significant (or marginally significant) after including the psychological drivers. Both autonomous and controlled motivation were significant predictors of goose hunting in the second step of the model, but autonomous motivation was no longer significant in step three when goose hunter identity had been added to the models. Comparable situational determinants were important in the first step of the analyses of the intention to increase goose hunting, and both controlled and autonomous motivation were associated with a stronger intention to increase hunting. However, in contrast to the other models, autonomous motivation remained significant in step 3. The explained variance in all models increased significantly after adding the psychological drivers.

**Table 4:** Situational factors, motivation, and goose hunter identity as predictors of goose hunting frequency, goose bag size, and intention to increase goose hunting among ‘Goose hunters’.

	Goose hunting frequency	Goose bag size	Intention to increase goose hunting
	(β)	(β)	(β)
*Step 1: Situational factors*			
Access owner (D)	− 0.03	− 0.10*	0.00
Access lease (D)	0.02	0.01	0.05
Access oral agreement (D)	0.13***	0.10*	0.13**
Access hunting guest (D)	− 0.05	0.02	− 0.07
Number of hunting grounds	0.23***	0.29***	0.11*
Distance to hunting ground	− 0.10*	0.01	− 0.07^a^
Dog trained for goose hunting (D)	0.17***	0.15***	0.21***
Family/friends are goose hunters	0.16***	0.10*	0.16***
***F*** **value**	20.37	15.14	14.49
**Adj R**^**2**^	0.21***	0.17***	0.16***
*Step 2: Situational factors and motivation*			
Access owner (D)	0.00	− 0.08^a^	0.07*
Access lease (D)	0.01	0.01	0.04
Access oral agreement (D)	0.11**	0.09*	0.06^a^
Access hunting guest (D)	− 0.05	0.01	− 0.07*
Number of hunting grounds	− 0.20***	0.26***	0.04
Distance to hunting ground	− 0.11**	0.00	− 0.10**
Dog trained for goose hunting (D)	0.15***	0.13***	0.14***
Family/friends are goose hunters	0.14***	0.09*	0.11***
Controlled motivation	0.15***	0.12**	0.11**
Autonomous motivation	0.14***	0.12**	0.46***
**F change**	20.32	12.36	111.46
**ΔR**^**2**^	0.05***	0.04***	0.24***
**Adj R**^**2**^	0.26***	0.20***	0.40***
*Step 3: Situational factors, motivation and goose hunter identity*			
Access owner (D)	0.01	− 0.08^a^	0.07*
Access lease (D)	0.00	0.00	0.04
Access oral agreement (D)	0.12***	0.09*	0.07^a^
Access hunting guest (D)	−0.01	0.04	-0.06
Number of hunting grounds	0.13***	0.22***	0.02
Distance to hunting ground	−0.09**	0.01	-0.09**
Dog trained for goose hunting (D)	0.10**	0.10**	0.12***
Family/friends are goose hunters	0.06	0.05	0.09*
Controlled motivation	0.12***	0.11**	0.10**
Autonomous motivation	−0.03	0.03	0.41***
Goose hunter identity	0.44***	0.24***	0.15***
**F change**	122.69	28.61	14.50
**ΔR**^**2**^	0.13***	0.04***	0.01***
**Adj R**^**2**^	0.39***	0.24***	0.41***

*D* dummy.

^a^*p* < 0.08, *p < 0.05, **p < 0.01, ***p < 0.001.

The mediation analyses (Table S4) showed that controlled motivation only displayed an indirect effect on frequency, but not on bag size and intention to increase goose hunting. Autonomous motivation had a significant indirect effect on frequency, bag size, and intention to increase hunting. The total effects models revealed that autonomous motivation had a stronger effect on the three dependent variables than controlled motivation even though all effects were significant. This result suggests that autonomous motivation was overall more important than controlled motivation. However, the effect was indirect for hunting frequency and bag size, but direct and indirect for intention to increase goose hunting.

## Discussion

A dilemma in flyway management of geese, and more generally in wildlife management of fast-growing populations causing ecosystem disservices, is when hunters’ efforts reach a threshold, resulting in a decreasing relative harvest rate and a continued undesired population growth^13,50,51^. Even though our study revealed an overall limited potential to increase goose hunting among hunters, it suggests ways to address this management dilemma by outlining structural, situational, and psychological factors important for goose hunting and to increase goose harvest.

In our study, having a goose hunting parent during childhood was associated with higher levels of involvement in goose hunting, confirming the importance of socialization revealed in previous research of hunters^20^. The proportional dominance of men among ‘Goose hunters’ is in line with goose hunter studies^28^. The most important characteristics associated with ‘Goose hunters’, as compared to the other groups, were a descriptive norm supporting goose hunting and a higher level of subjective knowledge about it. ‘Goose hunters’ further displayed beliefs that goose hunting is more exciting and less boring as well as better access to hunting grounds holding high numbers of geese than the other groups. Similar factors have been found to differentiate hunters from non-hunters in other contexts^17^. In addition, Vayer et al.^21^ suggest that lack of knowledge of hunting may be a particularly important constraint among potential future hunters. Contrary to previous studies, our study revealed that lack of time and available land for hunting were, together with material factors, less important for discriminating between the groups. Yet, the importance of material factors for goose hunting has been noted^28^ and poor access to land or equipment may still be significant barriers among non-goose hunters preparing to start hunting geese, given that concerns associated with e.g., feasibility tend to become more important as plans to act are formed^52^.

The majority of ‘Goose hunters’ (55%) had a goose bag size of five birds or less, which is comparable to a Danish study reporting a mean of six but a median of two geese^28^. In addition, 27% of the hunters in our study stated that they went goose hunting on average more than 10 days during a hunting season. In our data, situational determinants were positively associated with both goose hunting frequency and bag size, including greater access to hunting ground by oral agreement, access to more hunting grounds, a dog in the household trained for goose hunting, and more goose hunters among family and friends. The increase in explained variance after adding the psychological factors further supports their importance as predictors of goose hunting. Autonomous motivation had a stronger impact than controlled motivation on goose hunting, but the impact was via a goose hunter identity. Our results support the validity of integrating SDT^40^ with identity theory^42^ to understand psychological drivers of hunting, thus complementing the explicit motive approach employed in previous studies. The presented framework is validated in relation to both current goose hunting and future intention.

**Figure 3:**
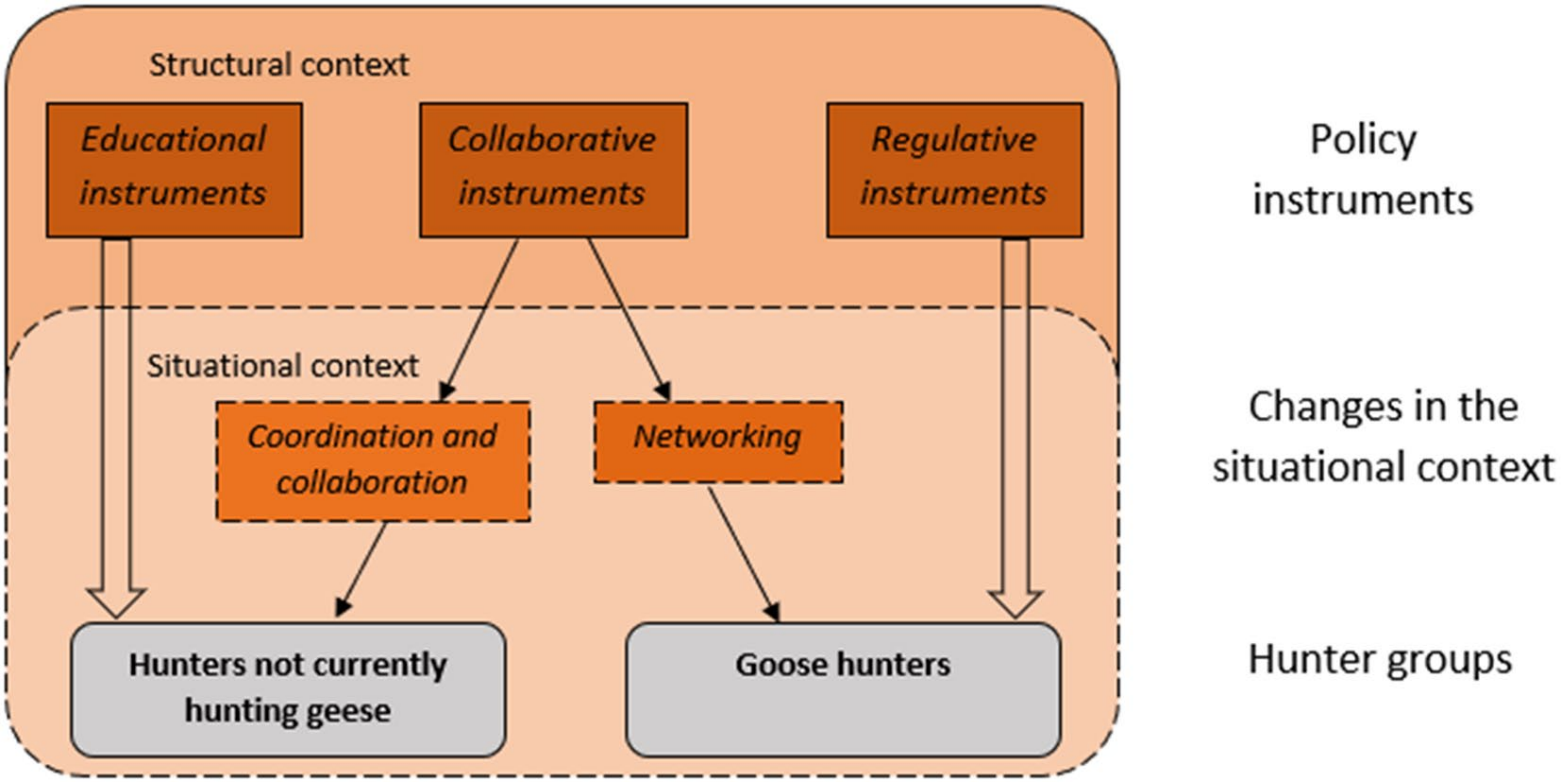
Policy instruments targeting different groups of hunters via changes in the situational context.

Our study provides insight on why harvest, or hunters’ effort, may not correspond to a continued population increase in geese^13,51^. It also adds to studies proposing general guidelines for international flyway management of geese^4^ by conveying practical suggestions for how to utilize policy instruments to target hunters as part of flyway management of geese in Europe and potentially also North America. This study suggests that it is easier to encourage goose hunters to intensify their goose hunting than to attract new hunters to start hunting geese. It also confirms that different instruments are needed when targeting different groups of hunters (Fig. 3). For example, extending the hunting season (a regulatory instrument) and financial instruments targeting hunters directly may intensify hunting among goose hunters. However, since financial instruments may crowd out autonomous motivation (i.e., more internalized form of motivation)^53^, financial resources may be better used to support hunters indirectly via the management system or hunters’ associations. In this study, educational instruments (i.e., goose hunting education) initiated a stronger response to increase goose hunting among ‘Non-goose hunters’ and ‘Goose hunters’ than among ‘Lapsed goose hunters’. Limited knowledge in both groups currently not involved in goose hunting indicates that educational instruments are likely most useful among these hunters.

Collaborative instruments may be used when targeting different groups of hunters, but in different ways (Fig. 3). This study revealed that ‘Non-goose hunters’ and ‘Lapsed goose hunters’ expected a greater increase in response to collaborative instruments than did ‘Goose hunters’, suggesting that the former groups are open towards such initiatives. Through e.g., coordination and collaborative hunts, goose hunting may become a more important part of the local hunting culture and the knowledge about goose hunting may increase. By encouraging ethically sound and environmentally sustainable goose hunts during these events (i.e., avoiding crippling of birds and lead ammunition contamination), the stewardship role of hunters may be strengthened, and additional benefits achieved^28,54^. Local management involvement in these events may be used to build ties, and subsequently trust, between management and local hunters, thus facilitating support for future management decisions (e.g., future shifts in hunting pressure). While hunters’ response to networks was not examined in this study, hunting may arguably be facilitated by strengthening local networks among current goose hunters, since these may facilitate oral agreements between hunters and landowners, thereby removing situational barriers associated with access to hunting grounds and potentially also to a retriever dog. As specialization is important for effective goose hunts^28^, sustaining networks are likely key to maintained goose hunting. To increase hunting, it is not only important to consider access and opportunities, but also the quality of the hunt^19^. Since autonomous motivation is associated with several psychological and social benefits (e.g., independence and confidence in skills, connections with others)^40^, encouraging autonomous motivation is likely to benefit the quality of hunts.

This study targeted a representative sample of hunters in the south of Sweden and respondents displayed a fairly good correspondence with the population of hunters. However, hunters interested in goose hunting are likely to be overrepresented among our respondents (two out of five were goose hunters). Questions were developed based on previous studies, yet there is a need to be cautious e.g., when interpreting the strength of intention to increase goose hunting in the future, since intentions are not always realized in practice^55^. In addition, responding hunters may not have considered all circumstances when estimating how they would react to a policy instrument. Nevertheless, our reliability assessments suggest that the indicators were largely reliable, and results provide an initial overview of the barriers and drivers for increased goose hunting. Interventions in the field, drawing on insights of behavioral interventions research^56^, and evaluating impacts on different types of measures (e.g., beliefs and emotions, official reports of hunting bags) would be important to improve the understanding of goose hunters’ effort and performance.

Overall, this survey study suggests that it may not be an easy task to increase goose hunting among Swedish hunters as part of the European flyway management, though we found a higher potential to do so among goose hunters. Goose hunters may e.g., be encouraged by prolonged hunting seasons and by facilitating local ties with landowners to potentially remove situational barriers. Other hunters may rather be reached via involvement in e.g., collaborative goose hunts to ensure a strengthening of a descriptive goose hunting norm and boosting knowledge levels. Supporting hunters’ internal motivation of goose hunting, rather than using external pressures, is likely to sustain goose hunting over time and is also needed to ensure appropriate specialization. Regulatory instruments are enforced at higher levels in the management system, but successful implementation of e.g., local collaboration, also requires resources and support^57,58^. Hence, multiple levels of the management system need to contribute to increase hunters’ involvement in the international flyway management of geese.

## Methods

### Study context

The goose species causing damage on agricultural land in Sweden are either breeding or staging/wintering birds. The highest densities of geese are found in the south of Sweden^59^. Natural foraging habitats include wetlands and coastal meadows but there is an increasing grazing pressure on agricultural land, frequently causing crop damage^60^. Goose management incorporates multiple levels of governing bodies and forums for participation, from the international and European levels (Agreement on the Conservation of African-Eurasian Migratory Waterbirds and European Goose management Platform) to the national, regional, and local levels in Sweden^58^. At the regional level, the County Administrative Boards (CABs) play an important role, e.g., by granting permission for derogation shooting (i.e. shooting of protected species or outside open hunting season for huntable species)^61^. Hunters are represented at all levels in the management system with local hunters needed as part of the implementation of flyway management of geese.

### Sample

A questionnaire was sent to a random sample of hunters (n = 5,000) (with an approved hunting exam and a purchased hunting license in 2021), 20–67 years of age in 13 counties in the south of Sweden (Skåne, Halland, Västra Götaland, Östergötland, Gotland, Jönköping, Kronoberg, Kalmar, Örebro, Västmanland, Uppsala, Södermanland, and Blekinge). The sample was drawn from the register of hunters at the Swedish Environmental Protection Agency after a confidentiality review. The net-sample comprised 4930 hunters and the response rate was 35.6% (n = 1753). An analysis of the attrition revealed that the distribution of respondents across counties did not differ from the overall sample (χ^2^ (12, 5000) = 11.55, *p* = 0.482), but respondents were older than the sample (50 compared to 45 years, t(4998) =  − 13.38, *p* = 0.001). Preliminary register information about the population of hunters in the selected counties showed no deviation in gender distribution (χ^2^ (1, 109 282) = 1.54, *p* = 0.214).

### Measures

The questionnaire was developed amongst the interdisciplinary team of authors, based on operationalizations of theoretical concepts (e.g.,^47,62^) and previous research^15,28^. Respondents answered questions about hunting behavior in general, structural characteristics, beliefs reflecting situational and psychological characteristics associated with goose hunting, and the potential to increase their goose hunting. Only respondents who answered that they had hunted geese at least once during the past five hunting seasons answered questions about their own goose hunting and determinants of goose hunting. The respondents were categorized into three groups based on questions about how long ago they first participated in a goose hunt as a hunter (including the response “have never participated”) and which hunting seasons they had participated in a goose hunt as a hunter (with the past five hunting seasons and “none of these years” as response categories). When two or more items were used to measure a variable, internal reliability was assessed using Cronbach’s alpha (α) with a value closer to 1 reflecting a higher consistency in the measure.

#### Hunting in general and structural characteristics

Two questions reflected hunting in general. One measured how long ago the respondent first participated in a hunt as a hunter (0–5 years ago, 6–10 years ago, 11–20 years ago, 21–30 years ago, 31 years or more) and one measured hunting behavior the previous season (0 days, 1–5 days, 6–10 days, 11–20 days, 21–50 days, more than 50 days)*.* Structural characteristics including growing up with a parent that went goose hunting (yes, no, don’t know), gender, age, education, and residency (from rural or less than 200 inhabitants to more than 100 000 residents) were measured.

#### Situational and psychological characteristics

Beliefs about goose hunting (five positive and seven negative) were measured reflecting hunters’ beliefs of how situational and psychological characteristics are associated with goose hunting^15,28^, (see also^17^). Answers were provided on a five-point scale (Completely disagree to Completely agree). We measured beliefs about psychological characteristics including having limited knowledge of goose hunting, and to what extent goose hunting was believed to be exciting and/or boring. Beliefs about situational characteristics covered dimensions associated with the ecosystem (i.e., there are too few geese) and material (i.e., easy access to hunting grounds, poor access to a retriever dog, and poor access to equipment). In addition, social dimensions reflecting descriptive goose hunting norms (i.e., goose hunting part of hunting culture, goose hunting is not prevalent where they live) and injunctive goose hunting norms (i.e., goose hunting is accepted among people around them, goose hunting is accepted in the general public) as well as personal dimensions (i.e., time consuming, demanding) were measured.

#### Potential for increased goose hunting

Intention to increase goose hunting was measured by means of four items regarding how likely it is that the respondent would regularly participate in goose hunting during the open hunting season and in derogation shooting of geese, shoot more geese than today, and more frequently go on goose hunts than today, utilizing a five-point response scale (Not at all likely to Very likely) (α = 0.88). Response to policy instruments was measured via the question: “How do you evaluate that the magnitude of your goose hunting would be influenced the coming five hunting years if the following instruments would be implemented?” using a five-point response scale (Reduce hunting, No change, Increase hunting). Two items each were used for the following instruments: Education (offers of education about goose hunting including practical and theoretical elements, offers of education about derogation shooting on birds, α = 0.92), Collaborative (the hunt is coordinated locally by e.g., the hunting team, the owner of hunting rights or the scaring consultant employed by the CAB, large collaborative hunts are arranged nearby, α = 0.76), and Financial (daily allowance for goose hunting (i.e., compensation for each hunting day) is introduced, it will be possible to request bounties for harvested geese, α = 0.90). In addition, the following instruments were examined: the possibility to borrow hunting equipment such as decoys from common resource pools, better access to game meat processing plants to simplify sale of hunted geese, and extended hunting season for abundant geese.

#### Goose hunting

Directed towards the ‘Goose hunters’ only, measures of goose hunting covered hunting frequency and bag size^28^. The number of hunting days, on average, during a) the open hunting season, b) derogation shooting based on own initiative (i.e., when permission is not required by the CAB), c) derogation shooting after permission by the CAB, d) derogation shooting of barnacle goose, and e) derogation shooting of other goose species (e.g., graylag goose, Canada goose) were asked about, with the possibility to answer one of the following six categories: 0 days, 1–5 days, 6–10 days, 11–20 days, 21–50 days, > 50 days. A measure of total hunting frequency per hunting season (ordinal scale) was created (e.g., response category 1 for both equals category 1, response category 2 for both equals category 2). Average bag size during a goose hunt (open hunting season and derogation shooting combined) were also measured with the following response five options: 0–2, 3–5, 6–10, 11–50, > 50 (including the option that they do not participate). A measure of total bag size was created (ordinal scale) (e.g., response category 1 for both equals response category 2, response category 1 and 4 equals 4).

#### Determinants of goose hunting

Several questions covered goose hunting specific situational factors including household access to a dog trained for goose hunting (yes/no), access to hunting ground (own, lease, hunting license, oral agreement, invited hunting guest, yes/no), number of hunting grounds (one, two, three, four or more), distance to most frequently used hunting ground (0–10 km, 11–40 km, 41–80 km, 81–160 km, > 160 km), and how many among their friends and family who are goose hunters (no one, a few, half, more than half, almost everyone) (cf.^28^). In addition, motivation and self-identity were measured. To measure different levels of internalized motivation for goose hunting, items were developed based on applications of SDT in the field of leisure behaviors^62^. Autonomous motivation and controlled motivation were measured by means of six items each (e.g., reflecting that they hunt geese because it is fun (more internalized motivation) or to get access to hunting grounds for other game (external motivation)) (Table S1). The internal reliability was good for measures of autonomous motivation (α = 0.81) but slightly low for controlled motivation (α = 0.65). Goose hunter identity was measured using one item: “To what extent would you describe yourself as a goose hunter?” and answers were provided on a five-point response scale (Not at all, A little, Partly, A lot, Completely) (cf.^47^).

### Procedures

The study was performed in accordance with the ethical standards as laid down in the 1964 Declaration of Helsinki. Before participation, the hunters were informed about the study (including how personal information was handled during the project). They were also informed that participation in the study is voluntary before consenting to take part in it. Only anonymous data were analysed. Since no sensitive personal information was collected (as defined in Swedish legislation, the Ethics Review Act 2003:460) no explicit ethical approval was required for this study. The survey was conducted in the spring of 2022 by Kvalitetsindikator AB, a survey company. Hunters were initially invited via postal letter to take part in the survey digitally and the questionnaire was subsequently distributed via postal mail. Respondents were given the opportunity to respond via mail or digitally. Two reminders were sent by mail (including one with a new questionnaire) and SMS reminders were sent to those with a publicly available phone number. The majority, 67%, responded to the survey digitally.

### Analyses

Analyses were conducted using the SPSS 28.0 statistical software. Based on questions on how long ago they first participated in a goose hunt as a hunter and whether they have hunted geese the past five hunting years, hunters were divided into three groups: ‘Non-goose hunters’ (n = 675, 43%) (i.e., those who never hunted geese), ‘Lapsed goose hunters’ (n = 278, 18%) (i.e., those who previously hunted geese but don’t anymore), and ‘Goose hunters’ (n = 606, 39%) (i.e., those who hunted geese at least one of the past five hunting years). Even though Likert scale measures generate ordinal scale data, parametric tests can be employed^63,64^. The sufficiently large sample size in our study further assures that parametric tests are applicable. Effect size was assessed by Cramér’s and Partial η^2^ using the guidelines proposed by^65^. First, the three groups were compared regarding hunting in general and structural characteristics using Chi square tests and Cramér’s V to calculate effect size, except for age for which a univariate ANOVA was conducted with post hoc tests (Bonferroni correction), and Partial η^2^ was used to calculate effect size. In addition, group differences in beliefs about goose hunting were examined by means of univariate ANOVAs with post hoc tests (Bonferroni correction) and Partial η^2^ to calculate effect size. Second, the potential for increased goose hunting was evaluated by comparing the intention of the groups to increase goose hunting and expected change in goose hunting in response to the policy instruments via univariate ANOVAs with post hoc tests (Bonferroni correction) and Partial η^2^ to calculate effect size.

Third, descriptive analyses of goose hunting (hunting frequency and bag size) and determinants were conducted among ‘Goose hunters’. To analyze drivers of goose hunting, three linear hierarchical regression analyses with situational determinants, motivation and goose hunter identity as predictors and hunting frequency, bag size, and intention to increase goose hunting as dependent variables, respectively, were conducted. In the first step, situational factors covering access to hunting grounds (as an owner, lease, oral agreement, and hunting guest, dummy variables), number of hunting grounds (ordinal), distance to hunting ground (ordinal), dog trained for goose hunting in household (dummy), and number of goose hunters among family/friends (ordinal) were analyzed. In the second step, controlled and autonomous motivation were added to the models, and in the third step, goose hunter identity was included. The indirect and total effects of controlled and autonomous motivation on goose hunting were analyzed via a mediation analysis of the psychological drivers^66^. A macro, MEDIATE [with a bootstrapping method (10 000 samples, 95% confidence interval)]^67^, was utilized to test mediation by means of an omnibus test of indirect effects and the total effects of controlled and autonomous motivation on the three indicators of goose hunting.

## Supplementary Information

Supplementary Information.

## Data Availability

The datasets generated and analyzed during the current study are available from the corresponding author on reasonable request.
