# Supplementary material for: The importance of structural, situational, and psychological factors for involving hunters in the adaptive flyway management of geese
Source: Sci Rep. 2023 May 2;13:7112. doi: 10.1038/s41598-023-33846-0 (PMC10154402; doi:10.1038/s41598-023-33846-0)
Supplement: Supplementary file 1 — Supplementary Information. [file 41598_2023_33846_MOESM1_ESM.pdf]

Supplementary Information

Table S1. Items used to measure autonomous and controlled motivation.

Autonomous motivation		
	Intrinsic	I enjoy goose hunting
		I get pleasure and satisfaction from participating in goose hunting
		I hunt geese because it’s fun
	Identified	It’s important for me to hunt geese regularly
		I think it is important to make the effort to hunt regularly.
		There are benefits with goose hunting that I value
Controlled motivation		
	Introjected	I feel guilty if I don’t hunt geese
		I feel ashamed if I miss a goose hunt
		I feel I should hunt geese when possible
	External	I hunt geese because others in the hunting team or neighbors say I should
		I feel under pressure from landowners to hunt geese
		I hunt geese to get access to hunting grounds for other game

Table S2. The average number of days spent on goose hunting during the hunting season (open hunting season, derogation shooting and in total) (goose hunting frequency) and average bag size on a goose hunt (open hunting season, derogation shooting, and in total) (goose bag size) (as percent for each category) among ‘Goose hunters’.

	Open hunting season	Derogation shooting	Total
Goose hunting frequency			
0 days	13	45	8
1-5 days	67	35	36
6-10 days	12	11	29
11 days or more	8	10	27
Goose bag size			
0-2	56	62	14
3-5	24	21	41
6-10	12	11	24
More than 11	8	6	21

Note. Despite being categorized as goose hunter, 8% stated that they hunted geese in total 0 days on average during the hunting season. The vast majority of these goose hunters, hunted only one of the last five hunting seasons, frequently a couple of years ago.

Table S3. Situational factors associated with goose hunting among ‘Goose hunters’.

		Frequency
Access to hunting land		
	Own	24%
	Lease	40%
	Hunting license	4%
	Oral agreement	41%
	Invited hunting guest	54%
Number of hunting grounds		
	One	38%
	Two	30%
	Three	17%
	Four or more	14%
Distance to most frequently used hunting ground		
	0-10 km	39%
	11-40 km	37%
	41-80 km	14%
	81-160 km	6%
	Longer than 160 km	4%
Hunting dog in household for goose hunting		23%
Family and friends goose hunters		
	No one	15%
	A few	65%
	Half	12%
	More than half	6%
	Almost everyone	2%

Table S4. Indirect effects and total effects of controlled and autonomous motivation on goose hunting frequency, goose bag size, and intention to increase goose hunting among 'Goose hunters'.

	Goose hunting frequency		Goose bag size		Intention to increase goose hunting	
Indirect effects (via goose hunter identity)	ci 95%		ci 95%		ci 95%	
Controlled motivation	0.009 – 0.191		-0.005 – 0.087		-0.003 – 0.069	
Autonomous motivation	0.276 – 0.431		0.114 – 0.2183		0.084 – 0.173	
Total effects	Coeff(s.e)	t	Coeff(s.e)	t	Coeff(s.e)	t
Controlled motivation	0.359 (0.092)	3.924***	0.249 (0.074)	3.362***	0.209 (0.064)	3.253**
Autonomous motivation	0.356 (0.061)	5.849***	0.253 (0.050)	5.058***	0.604 (0.043)	14.162***

***p <.001.

Note. When the confidence interval (ci) includes 0, the effect is not significant [67].
